# Sensory symptom profiles differ between trigeminal and thoracolumbar postherpetic neuralgia

**DOI:** 10.1097/PR9.0000000000000636

**Published:** 2018-02-06

**Authors:** Stefanie Rehm, Moritz Groβkopf, Maria Kabelitz, Thomas Keller, Rainer Freynhagen, Thomas R. Tölle, Ralf Baron

**Affiliations:** a Sektion Neurologische Schmerzforschung und –therapie, Klinik für Neurologie Universitätsklinikum Schleswig-Holstein, Kiel, Germany; b StatConsult GmbH, Magdeburg, Germany; c Zentrum für Anästhesiologie, Intensivmedizin, Schmerzmedizin und Palliativmedizin, Benedictus Krankenhaus Tutzing, Tutzing, Germany; d Klinik für Anästhesie, Technische Universität München, München, Germany; e Klinik für Neurologie, Technische Universität München, München, Germany

**Keywords:** Neuropathic pain, Postherpetic neuralgia, Cephalic/extracephalic

## Abstract

**Introduction:**

Animal experimental evidence suggests that mechanisms of pain generation and response to treatment differ between neuropathic pain in the cephalic and the extracephalic innervation territories.

**Objectives:**

The objective of the study was to examine whether in humans an identical peripheral painful neuropathy is associated with different pain qualities and sensory abnormalities in the face as compared with the thoracic region.

**Methods:**

We retrospectively analysed epidemiological and clinical data of 639 patients with postherpetic neuralgia (PHN) in the face and at the trunk who were collected within a cross-sectional cohort survey and compared the respective sensory symptom profiles captured with the painDETECT questionnaire.

**Results:**

Two hundred twenty-four patients suffered from trigeminal PHN and 415 from thoracolumbar PHN. There were no significant differences in sex-ratio, age, body mass index, and pain duration. Patients with trigeminal PHN were more often severely depressed. Anxiety and sleep scores were not different. The average pain intensity was slightly higher in thoracolumbar PHN than trigeminal PHN (visual analogue scale 5.0 vs 4.6). Postherpetic neuralgia in the thoracolumbar region showed significantly more intense burning sensations, allodynia, painful attacks, and significantly less prickling and numbness than PHN in the face.

**Conclusions:**

The differences in sensory symptom profiles between facial PHN and truncal PHN might be associated with different pathophysiological mechanisms and different treatment response. Drugs that primarily act on sensitization processes in the peripheral nociceptive system may work better in thoracolumbar PHN than in trigeminal PHN. If new medications are tested in patients with PHN, it would therefore be of interest to include an analysis of the treatment results in regard to subgroups based on the localisation of pain in patients with PHN.

## Abstract 2

Differences in somatosensory profiles in different localisations in 1 distinct disease (postherpetic neuralgia) were shown. This might have implications for future research and treatment regimes.

## 1. Introduction

Neuropathic pain, ie, pain which occurs as a direct consequence of a lesion or disease of the somatosensory system^6^ is frequent and often difficult to treat. In the periphery, every afferent nerve of the body can be affected. There is, however, animal experimental evidence that mechanisms of pain generation and response to treatment differ between the cephalic and the extracephalic innervation territories.^4,11,13,18^

To translate this body area–related discrepancy into pain mechanisms to human pain states, we investigated whether an identical peripheral painful neuropathy, ie, postherpetic neuralgia (PHN), is associated with different pain qualities and sensory abnormalities in the face as compared to the thoracic region. The sensory phenotype of patients with neuropathic pain can be used to speculate about different underlying mechanisms of pain generation in both groups.^3^

We analysed epidemiological and clinical data of more than 600 patients with PHN in the face and at the trunk who were collected within a cross-sectional cohort survey in Germany (pain*DETECT*) performed in collaboration with the German Research Network on Neuropathic Pain (DFNS).

## 2. Methods

### 2.1. Study population and data collection

The objective of the study was to examine whether an identical peripheral painful neuropathy, in this case PHN, is associated with different pain qualities and sensory abnormalities in the face as compared to the thoracic region and if so, whether this is due to a difference of the underlying pathophysiological mechanism.

The study was performed at 919 outpatient centers in Germany. Due to the fact that different practitioners (general practitioners, rheumatologists, orthopaedist, and pain specialists) participated in this study, not each 1 of the 919 centers could recruit patients for the study. Therefore, a total number of 639 patients were included. Patients presenting with PHN, at least 18 years old, used a hand-held computer to complete electronic patient-reported questionnaires for the cross-sectional epidemiological and clinical survey. The study protocol was approved by the ethical committee of the University of Düsseldorf.

The patient selection was retrospectively performed based on electronic pain drawings performed by the patients. The hand-held computer is equipped with a body drawing with 34 predefined body areas. Patients with PHN (pain >3 months after acute shingles) who marked their major pain in the trigeminal area or in the thoracolumbar dermatomes were included in the study.

To assess the somatosensory symptoms of the patients, the electronic version of the pain*DETECT* questionnaire (PD-Q^7,8^) was used (Table 1). The patients could rate the perceived severity of each symptom from 0 to 5 (never, hardly noticed, slightly, moderately, strongly, and very strongly). In detail, the questions address the following sensory symptoms: question 1—spontaneous burning pain, question 2—spontaneous prickling sensations, question 3—pain evoked by light touch (allodynia), question 4—spontaneous pain attacks, question 5—pain evoked by thermal stimuli, question 6—numbness, and question 7—pressure pain.

**Table 1:**
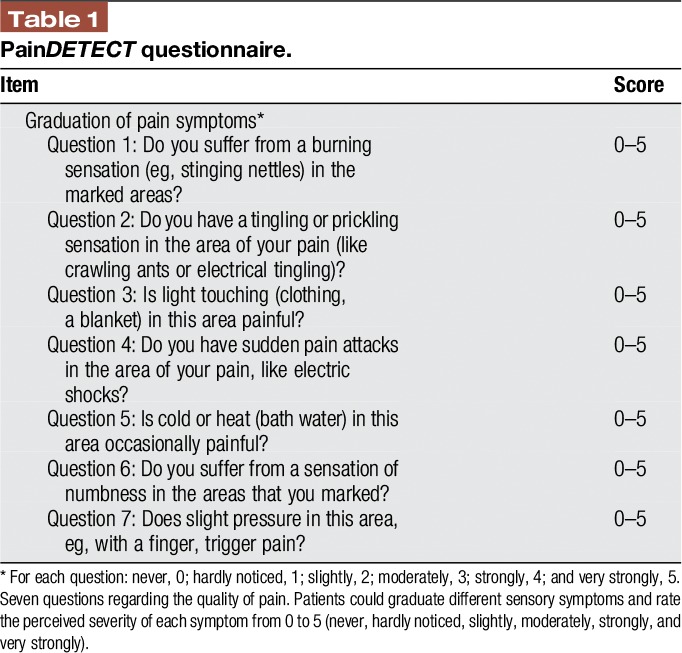
Pain*DETECT* questionnaire.

**Figure 1.**
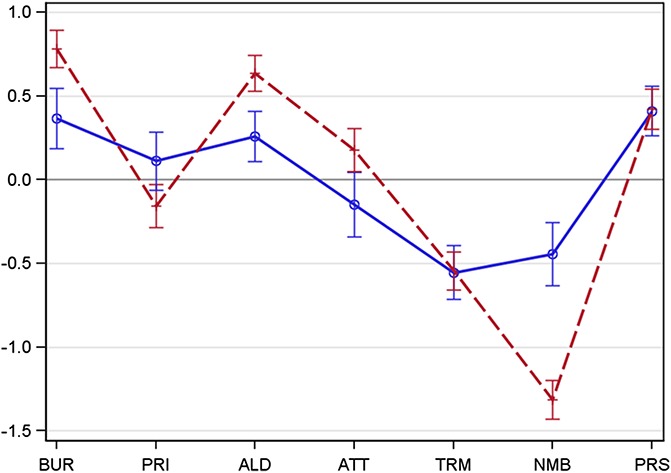
Spaghetti plot representing the distribution of somatosensory symptoms—face vs thoracic (adjusted). Intensity of sensory symptoms captured with the painDETECT questionnaire in trigeminal postherpetic neuralgia (blue, solid line, n = 277) and in thoracolumbar postherpetic neuralgia (red, broken line, n = 517). Mean + 95% CI. The symptom intensity is represented by the patterns of questionnaire scores (adjusted individual mean), thus showing the typical pathological structure of the respecting group. Sensory profiles show remarkable differences in the expression of the symptoms. Significant differences for burning, allodynia and attacks (thoracolumbar > trigeminal) as well as prickling and numbness (trigemial > thoracolumbar). ALD, allodynia; ATT, attacks; BUR, burning; CI, confidence interval; NMB, numbness; PRI, prickling; PRS, pressure; TRM, thermal.

Two calculations were performed: (1) To eliminate interindividual differences of the general perception of sensory stimuli (differences individual perception thresholds), a score was calculated in which the given 0 to 5 score of each question was subtracted by the mean of all values marked in the 7 questions. In this individual score, values above 0 indicate a sensation which is more intense than the individual mean perception and values below 0 indicate a sensation which is less intense than the individual mean perception^12^ (Figure 1 and Table 2). (2) The absolute values for each symptom intensity score were assessed and compared between the 2 subgroups.

**Table 2:**
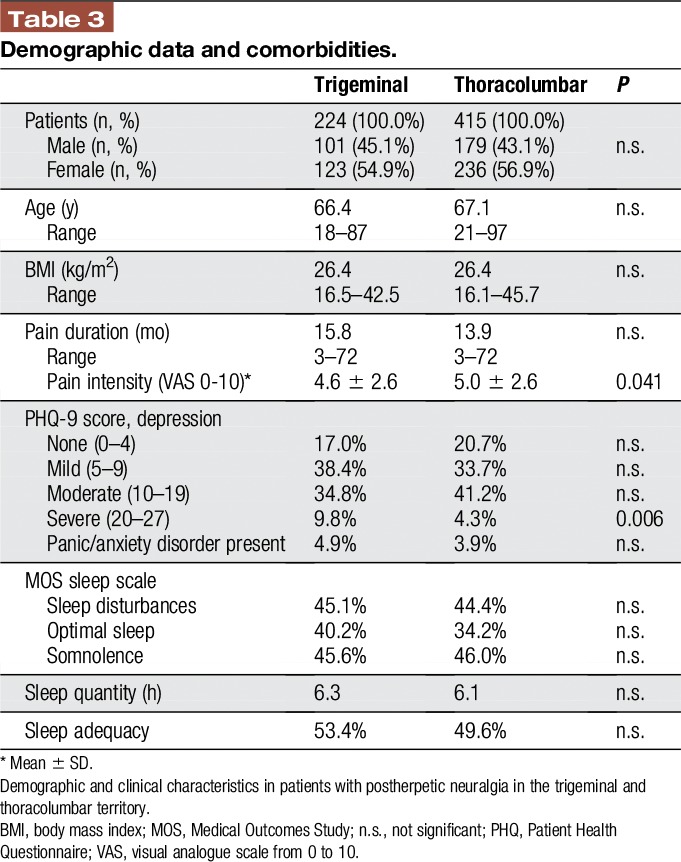
Distribution of somatosensory symptoms—face vs thoracic (adjusted).

In addition to standard demographic questions, the following validated questionnaires were used to assess comorbidities: for sleep disturbances, the Medical Outcomes Study sleep scale (MOS^9^) and for depressive disorders and panic and anxiety disorders, the German-language Patient Health Questionnaire (PHQ, short form^14^).

### 2.2. Statistics

Descriptive statistical analyses were performed. For each patient, a score was calculated in which the given 0 to 5 score of each question was subtracted by the mean of all values marked in the 7 questions to eliminate individual perception differences for sensory stimuli. Continuous variables were presented within tables by mean plus/minus SD, with 95% confidence intervals or ranges. Differences were evaluated for statistical difference by the 2-sample *t* test (2 sided, α level 0.05).

## 3. Results

### 3.1. Epidemiological features and comorbidities

Complete data sets from 639 patients with PHN were available for further analysis (Table 3), 224 patients suffered from trigeminal PHN and 415 from thoracolumbar PHN. There were no statistically significant differences in sex-ratio, age, body mass index, and pain duration. Patients with trigeminal PHN were more often severely depressed. Anxiety and sleep scores were not different in both groups.

**Table 3:**
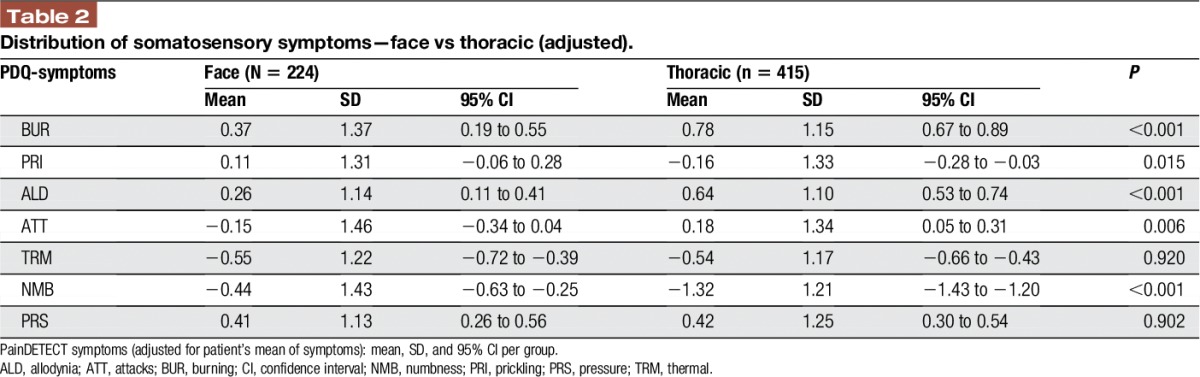
Demographic data and comorbidities.

### 3.2. Pain intensity and frequency of sensory symptoms

**Figure 2.**
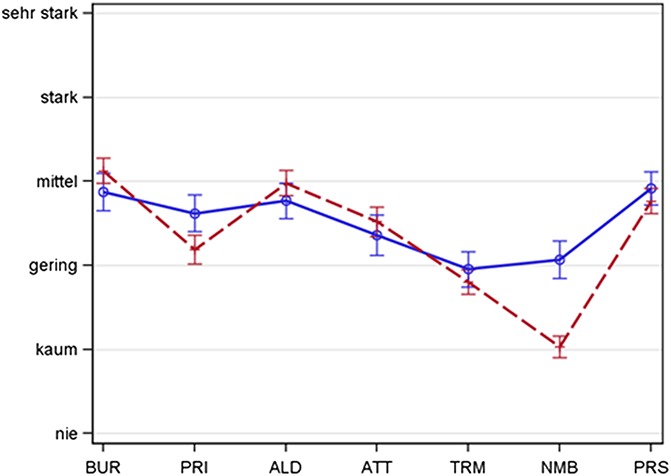
Spaghetti plot representing the distribution of somatosensory symptoms—face vs thoracic (not adjusted). Intensity of sensory symptoms captured with the painDETECT questionnaire in trigeminal postherpetic neuralgia (blue, solid line, n = 277) and in thoracolumbar postherpetic neuralgia (red, broken line, n = 517). Mean + 95% CI. The symptom intensity is represented by the patterns of questionnaire scores (not adjusted individual mean), thus showing the typical pathological structure of the respecting group. Sensory profiles show differences in the expression of the symptoms. Significant differences were shown for prickling and numbness. Patients could graduate different sensory symptoms and rate the perceived severity of each symptom from 0 to 5 (never = nie, hardly noticed = kaum, slightly = gering, moderately = mittel, strongly = stark, and very strongly = sehr stark). ALD, allodynia; ATT, attacks; BUR, burning; CI, confidence interval; NMB, numbness; PRI, prickling; PRS, pressure; TRM, thermal.

The average pain intensity was slightly higher in thoracolumbar PHN as compared to trigeminal PHN (visual analogue scale 5.0 ± 2.6 vs 4.6 ± 2.6; *P* value 0.041). The total painDetect score did not differ significantly between the 2 groups (thoracolumbar PHN 17.24 ± 7.20, trigeminal PHN 17.83 ± 7.24; *P* value 0.320). The intensity of the sensory symptoms was remarkably different between both groups. Postherpetic neuralgia in the thoracolumbar region showed significantly less prickling and numbness than PHN in the face (Figure 2 and Table 4). When the PDQ symptoms were adjusted to the individual mean, PHN in the thoracolumbar region also showed significantly more intense burning sensations, allodynia and painful attacks as well as significantly less prickling and numbness than PHN in the face (Figure 1 and Table 2).

**Table 4:**
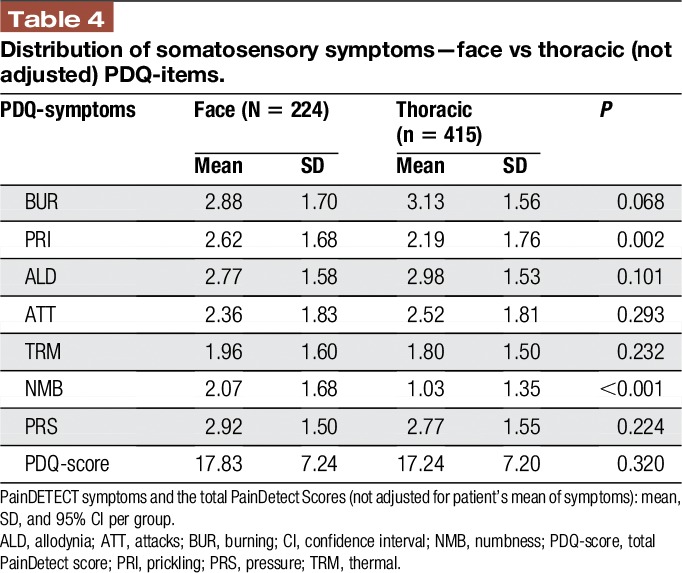
Distribution of somatosensory symptoms—face vs thoracic (not adjusted) PDQ-items.

## 4. Discussion and conclusion

In patients with PHN, pain qualities and sensory symptoms are different if the trigeminal nerve is affected or spinal nerves in the thoracolumbar territory. First, patients with PHN in the trunk suffer from more intense burning. The burning quality of neuropathic pain is believed to be associated with sensitization of heat-sensitive C-nociceptors, thus the burning is indicative of sensitization processes in primary afferent nociceptors.^2^ Second, allodynia was present more frequently in the trunk than in the face. Dynamic mechanical allodynia develops if mechanosensitive A-beta fibers activate sensitized second-order neurons in the spinal cord, thus allodynia is indicative of central sensitization.^20^ Third, painful attacks occur more often in the trunk than in the face. Short-lasting painful attacks are perceived if bursts of discharges mainly in nociceptive neurons are conveyed to the central nervous system.^1^ Fourth, numbness is nearly absent in the trunk as compared to the face. Numbness is regarded as a negative sensory symptom that points to a loss of afferent functions.^19^ Thus, the trunk is rather characterized by preserved afferent innervation, whereas the face shows more signs of nerve degeneration.

Taken these results together, PHN in the thoracolumbar body region demonstrates more signs of sensitization in relatively intact afferent neuronal systems than PHN in the face.

According to these results, animal experiments suggest that there are fundamental pathophysiological differences between pain syndromes caused by an injury to the trigeminal nerve as compared to a lesion of other peripheral nerves. In rats, Tal and Devor^18^ studied pathophysiological properties of injured afferent axons in the infraorbital nerve and in the sciatic nerve. Ongoing discharge and mechanosensitivity of myelinated and unmyelinated axons were much less frequently observed in the infraorbital nerve than in the sciatic nerve. Furthermore, no injury-induced sympathetic sprouting into the trigeminal ganglion could be demonstrated after trigeminal lesion which is a common phenomenon in dorsal root ganglia after sciatic nerve injury.^4^ Chronic constriction injury (CCI) to the sciatic nerve in rats induced an overexpression of the proinflammatory cytokine IL-6 and subsequent microglia activation in the dorsal horn. This mechanism is believed to be involved in the development of central pain hypersensitivity. By contrast, no such upregulation could be found in the spinal nucleus of the trigeminal nerve after trigeminal CCI.^13^ In addition, differential treatment effects on neuropathic pain behavior were shown in the cephalic vs the extracephalic territories.^16^ Triptans and calcitonin gene-related peptide receptor antagonists alleviated pain behavior caused by CCI to the infraorbital nerve but not the sciatic nerve in rats.^11,15,17^

There are some limitations of the study. The results of this study refer to patients suffering from PHN, a peripheral neuropathic pain condition. At this point, it is not known if these results can be transferred to other neuropathic conditions, especially to central neuropathic pain. Another limitation of the study is that there was no control for the analgesic medication the participating patients were taking. Accordingly, an influence of analgesics on the somatosensory symptoms described by the patients cannot be ruled out completely. Other studies using data of large cohorts of patients suffering from neuropathic pain were facing the same problems and we know that the influence of the medication on different subgroups of patients cannot be crucial because it has not been shown that a majority of patients (>50%) was treated with the same drug or the same drug combination.^3^ Furthermore, it has to be kept in mind that these are overall results and that individual cases can naturally present with different sensory profiles.

The differences in sensory symptom profiles and potentially also in pathophysiological mechanisms between facial PHN and truncal PHN might have implications for the interpretation and design of clinical trials in this indication. It is very well conceivable that drugs that primarily act on sensitization processes in the nociceptive system may work better in thoracolumbar PHN than in trigeminal PHN.^10^ Facing future studies, it would therefore be interesting to include an analysis of the treatment results in regard to subgroups based on the localisation of pain in patients with PHN.^5^

## Disclosures

S. Rehm has received speaking fees from Grünenthal GmbH, Bayer Vital GmbH and Pfizer and travel support from Astellas. M. Großkopf has received travel support from Sanofi-Genzyme GmbH. R. Freynhagen reports research support, personal consulting, or lecture fees in the past 2 years from Astellas, Develco Pharma, Galapagos, Grünenthal, Lilly, Merck, Mitsubishi Tanabe Pharma, and Pfizer. T. Tölle has given lectures and/or participated in advisory boards on behalf of Astellas, Grünenthal, Ely-Lilly, Pfizer, Mundipharma, Teva, Nevro, Angelini, and Dompe. R. Baron has received grants/research support from Pfizer, Genzyme GmbH, Grünenthal GmbH, and Mundipharma. He is a member of the EU Project No 633491: DOLORisk. A member of the IMI “Europain” collaboration (grant agreement 115007) and industry members of this are: AstraZeneca, Pfizer, Esteve, UCB-Pharma, Sanofi Aventis, Grünenthal GmbH, Eli Lilly, and Boehringer Ingelheim Pharma GmbH&Co.KG.

German Federal Ministry of Education and Research (BMBF): Member of the ERA-NET NEURON/IM-PAIN Project (01EW1503). German Research Network on Neuropathic Pain (01EM0903), NoPain system biology (0316177C). German Research Foundation (DFG). He has received speaking fees from Pfizer, Genzyme GmbH, Grünenthal GmbH, Mundipharma, Sanofi Pasteur, Medtronic Inc. Neuromodulation, Eisai Co Ltd, Lilly GmbH, Boehringer Ingelheim Pharma GmbH&Co.KG, Astellas, Desitin, Teva Pharma, Bayer-Schering, MSD GmbH, and Seqirus. He has been a consultant for Pfizer, Genzyme GmbH, Grünenthal GmbH, Mundipharma, Allergan, Sanofi Pasteur, Medtronic Inc. Neuromodulation, Eisai Co Ltd, Lilly GmbH, Boehringer Ingelheim Pharma GmbH&Co.KG, Astellas, Novartis, Bristol-Myers-Squibb, Biogenidec, AstraZeneca, Merck, Abbvie, Daiichi Sankyo, Glenmark Pharmaceuticals, Seqirus, Teva Pharma, Genentech, Galapagos NV, and Kyowa Kirin GmbH. The remaining authors have no conflicts of interest to declare.

This research was supported by a grant from Pfizer Germany without restriction on publication.
